# Pilot Implementation of a Telehealth Nurse Care Management Model for Rural Primary Care Patients With Chronic Pain

**DOI:** 10.1093/geroni/igaf122.2124

**Published:** 2025-12-31

**Authors:** Elise Hoffman, Sebastian Tong, Brennan Keiser, Basia Belza, Kushang Patel

**Affiliations:** University of Washington, Seattle, Washington, United States; University of Washington, Seattle, Washington, United States; University of Washington, Seattle, Washington, United States; University of Washington, Seattle, Washington, United States; University of Washington, Seattle, Washington, United States

## Abstract

Prevalence of chronic pain is higher in rural areas than in urban areas. Rural residents have limited access to evidence-based chronic pain care, including non-pharmacologic treatments. Healthcare shortages, a lack of specialty care services, and aging populations in rural areas place a high demand for pain management on primary care providers. To help address this challenge, we implemented a telehealth nurse care management model that integrates care coordination, cognitive behavioral therapy, and referral to a tele-exercise program for rural primary care patients with chronic pain. We partnered with two rural-serving health systems in Northeast Washington and Central North Carolina to pilot the intervention. We trained two care managers to deliver the intervention to patients for 6 months. Among 31 participants who were enrolled, the mean age was 57.2 years (SD = 11.9), 20 (65%) were female, 23 (74%) had an annual household income <$50,000, and 26 (84%) were not employed. The mean baseline pain intensity and interference (0-10 rating) score was 6.8 (SD = 1.8). Exit interviews with participants, care managers, and local champions will be completed in March-April 2025. Data analysis will be guided by the COM-B Model for Behavior Change and Framework for Reporting Adaptations and Modifications (FRAME). We will present the patient-reported outcome results from the pilot study, as well as qualitative data on the experience of rural patients and nurse care managers with the intervention. Lastly, we will discuss adaptations made to the intervention in preparation for a larger pragmatic trial in 416 patients from 4-5 rural-serving health systems.

